# Complete genome sequence of *Listeria seeligeri* strain 43-1 isolated from a Danish forest freshwater swamp

**DOI:** 10.1128/mra.00311-24

**Published:** 2024-06-12

**Authors:** Taya Tang, Jørgen J. Leisner

**Affiliations:** 1 Department of Veterinary and Animal Sciences, Faculty of Health and Medical Sciences, University of Copenhagen, Frederiksberg, Denmark; DOE Joint Genome Institute, Berkeley, California, USA

**Keywords:** *Listeria seeligeri*, freshwater, virulence factors

## Abstract

Here, we report the genome sequence of *Listeria seeligeri* 43-1 isolated from a Danish freshwater swamp using Oxford Nanopore sequencing. The isolate shared a high genomic similarity to two other *L. seeligeri* isolates from soil and water.

## ANNOUNCEMENT

We examined the presence of *Listeria* spp. in 22 Danish freshwater habitats while mapping the distribution of lactic acid bacteria (1). One milliliter of water sample was cultured in 9 ml of Half Fraser Broth (Thermo Scientific, USA) at 30°C overnight and subsequently on RAPID’L.mono medium (Bio-Rad, USA) at 37°C for 1–2 days. Colonies were subcultured on Tryptone Soya Agar (Oxoid, Thermo Scientific, USA) with 5% bovine blood at 37°C for 1 day and identified by matrix-assisted laser desorption/ionization-time of flight mass spectrometry (1). Compared to other studies (2–4), the frequency of *Listeria* spp. was low with only one isolate of *Listeria seeligeri*, commonly associated with the natural environment (5–7), detected at one of the 22 locations, a forest swamp (Table 1). The DNA was extracted from a single colony using DNeasy PowerSoil Pro Kit (Qiagen, Germany). DNA concentration and purity were measured with the Qubit dsDNA HS Assay kit and NanoDrop One (Thermo Fisher Scientific, USA). DNA size distribution was performed using Genomic DNA ScreenTapes on Agilent Tapestation 4200 (Agilent, USA). A barcoded SQK-NBD114.96 DNA library was prepared, loaded onto a primed FLO-PRO114M (R10.4.1) flow cell, and sequenced on PromethION P24, running MinKNOW Release 23.11.4 (Oxford Nanopore Technologies). Signal data were basecalled with Dorado basecall server v. 7.2.13 (Oxford Nanopore Technologies). Porechop v. 0.2.4 was used for adapter trimming, and Nanoq v. 0.10.0 (8) was used for removing reads below 1,000 bp and Q20. Draft *de novo* assembly was generated with Flye v. 2.9.3-b1797 (9–11) , setting the parameters meta and extra-params min_read_cov_cutoff = 10. The draft assembly was subsequently polished twice with Medaka v. 1.11.3 (Oxford Nanopore Technologies). Assembly graphs were inspected with Bandage v. 0.8.1 (12). Platon v. 1.7 (13) was used to classify plasmids. Contig mapping quality and coverage were assessed using Samtools v.1.19.2 (14). Contigs with a mapping quality <30 or a coverage <20 were removed with GNU AWK. Completeness and contamination levels were assessed using CheckM2 v. 1.2.2 (15, 16). Genome chimerism was evaluated using GUNC v. 1.0.6 (17). Genome was classified with GTDBtk v. 2.3.2 against the Genome Taxonomy Database (release 214) (18, 19). The average nucleotide identity (ANI) values between the 43–1 genome and 15 *L. seeligeri* genomes retrieved from GenBank were calculated by OrthoANIu tool (20) and visualized using Chiplot (https://www.chiplot.online/). Genome annotation was performed with PGAP (21–23). Virulence and bacteriocin-encoding genes were identified by the Virulence Factor Database (VFDB) (24), AntiSMASH 6.0 (25) and BAGEL 4 (26). All bioinformatic tools utilized default parameters unless specified otherwise.

**TABLE 1:** Sampling location description and genome characteristics for *L. seeligeri* 43–1

Description of location	
Location	Copenhagen, Northern Suburb, Geels Forest
Time	22 March 2021
Type	Edge of mosaic forest bog/swamp/fen
pH	6.09
Geographic coordinates	55°48′0.3″N 12°29′4.5″E
Sequencing statistics	
Number of raw reads (Mbp)	5,015
Raw-read N50 (bp)	11,676
Quality score of raw reads	20.9
Number of filtered reads (Mbp)	1,756
Filtered-read N50 (bp)	11,436
Quality score of filtered reads	23.5
Genome statistics (after quality filtering)	
Assembly size (bp)	2,880,240
Number of circular contigs	3
Contig N50 (bp)	2,795,563
Contig L50	1
GC (%)	37.5
Genome coverage	602×
Number of 5S rRNA	7
Number of 16S rRNA	7
Number of 23S rRNA	7
Number of tRNAs	68
Total number of genes	2,846
Total number of CDS	2,753
Total number of pseudogenes	34
Completeness (%)^*a*^	99.57
Contamination (%)^*b*^	0.08
GTDB Taxonomy^*c*^	*L. seeligeri*
Bacteriocins	None
Potential virulence factors	LIPI-1, internalins
Data accession	
BioProject	PRJNA1089104
BioSample	SAMN40526393
Genome assembly^*d*^	GCA_039556175.1
SRA^*e*^	SRR28374924

^*a*^ Completeness (%) is the estimated genome completeness based on the presence and/or absence of essential lineage-specific marker genes.

^*b*^ Contamination (%) is the estimated genome contamination based on the presence of multiple single-copy marker genes.

^*c*^ GTDB Taxonomy denotes the highest taxonomy assigned to specific genomes when classified against the Genome Taxonomy Database (GTDB).

^*d*^ The quality-filtered genome assembly has been deposited in GenBank at NCBI with each contig assigned a unique accession number: CP148897, CP148898, CP148899.

^*e*^ The raw sequencing data (before quality filtering) has been deposited in the Sequence Read Archive at NCBI.

**Fig 1:**
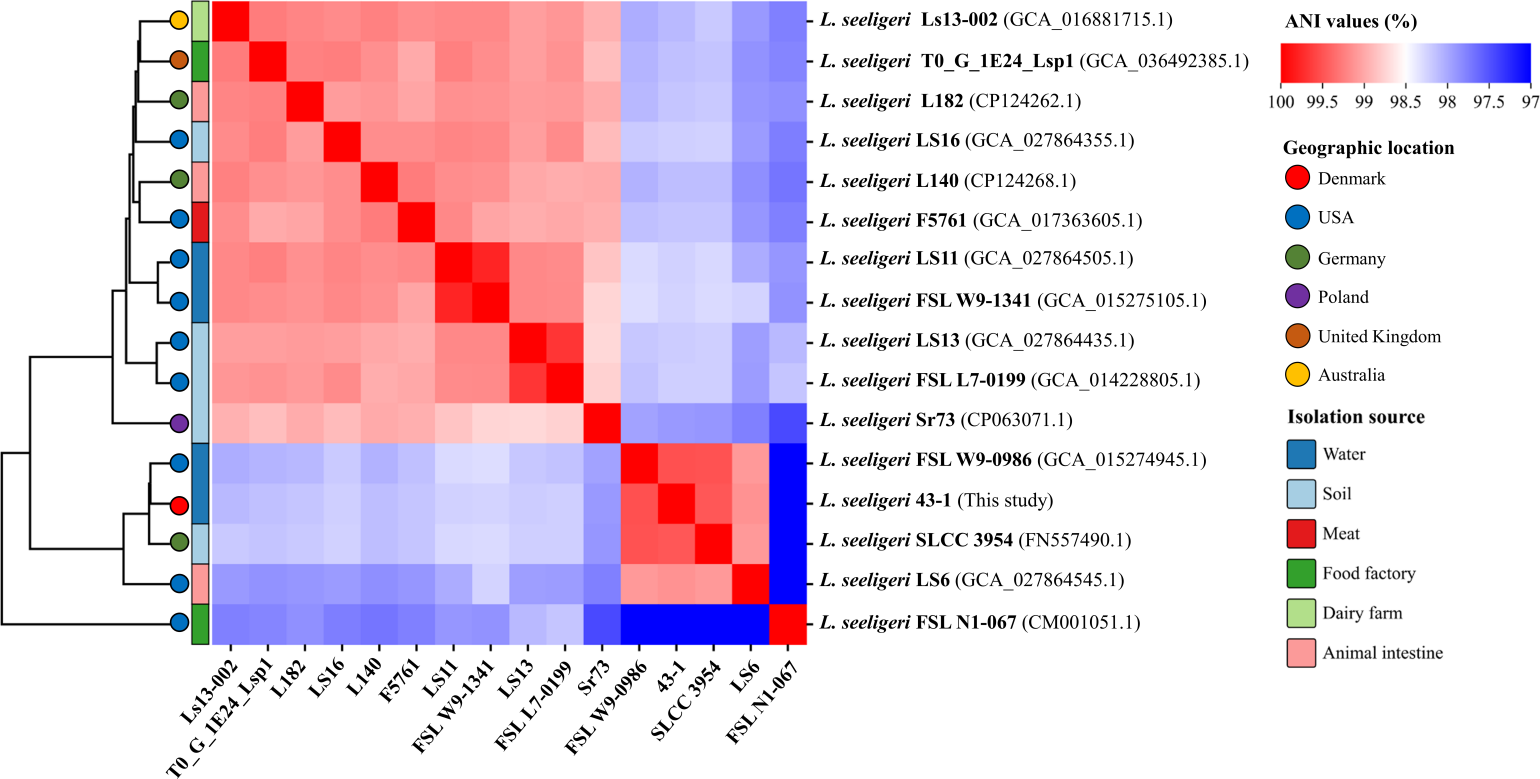
Heatmap of pairwise ANI between sixteen *L. seeligeri* genomes from various sources, including strain 43–1 from this study and 15 reference strains from GenBank. The dendrogram on the left of the heatmap was generated based on hierarchical clustering of the ANI values using Euclidean distance and average linkage.

The genome was assembled into three circular contigs, including a main chromosome (2,795,563 bp), a plasmid (79,543 bp), and a mobile genetic element from fragmented reads of the rRNA operons (5,134 bp) (Table 1). Isolate 43–1 shared high genomic similarity with FSL W9-0986 and SLCC 3954 from water and soil, with OrthoANI values of 99.53% and 99.49%, respectively, and less similarity with other strains from various sources (Fig. 1). The genome contained a Listeria Pathogenicity Island LIPI-1 but no genes encoding bacteriocins.

## Data Availability

Raw sequence reads have been deposited at the NCBI under BioProject with accession PRJNA1089104, BioSamples with accession SAMN40526393, and SRA with accession SRR28374924. The assembled genome has been deposited at GenBank, with accession GCA_039556175.1, and each contig assigned the accession number CP148897, CP148898 and CP148899, respectively.
